# Automated system for lung nodules classification based on wavelet feature descriptor and support vector machine

**DOI:** 10.1186/s12938-015-0003-y

**Published:** 2015-02-12

**Authors:** Hiram Madero Orozco, Osslan Osiris Vergara Villegas, Vianey Guadalupe Cruz Sánchez, Humberto de Jesús Ochoa Domínguez, Manuel de Jesús Nandayapa Alfaro

**Affiliations:** Departamento de Ingeniería Industrial y Manufactura, Instituto de Ingeniería y Tecnología, Universidad Autónoma de Ciudad Juárez, Av. del Charro 450 norte, Z. C. 32310 Ciudad Juárez, Chihuahua México; Departamento de Ingeniería Eléctrica y Computación, Instituto de Ingeniería y Tecnología, Universidad Autónoma de Ciudad Juárez, Av. del Charro 450 norte, Z. C. 32310 Ciudad Juárez, Chihuahua México

**Keywords:** CADx system, Lung nodules, CT scan, Wavelet feature descriptor, Gray level co-ocurrence matrix, Support vector machine, Texture

## Abstract

**Background:**

Lung cancer is a leading cause of death worldwide; it refers to the uncontrolled growth of abnormal cells in the lung. A computed tomography (CT) scan of the thorax is the most sensitive method for detecting cancerous lung nodules. A lung nodule is a round lesion which can be either non-cancerous or cancerous. In the CT, the lung cancer is observed as round white shadow nodules. The possibility to obtain a manually accurate interpretation from CT scans demands a big effort by the radiologist and might be a fatiguing process. Therefore, the design of a computer-aided diagnosis (CADx) system would be helpful as a second opinion tool.

**Methods:**

The stages of the proposed CADx are: a supervised extraction of the region of interest to eliminate the shape differences among CT images. The Daubechies db1, db2, and db4 wavelet transforms are computed with one and two levels of decomposition. After that, 19 features are computed from each wavelet sub-band. Then, the sub-band and attribute selection is performed. As a result, 11 features are selected and combined in pairs as inputs to the support vector machine (SVM), which is used to distinguish CT images containing cancerous nodules from those not containing nodules.

**Results:**

The clinical data set used for experiments consists of 45 CT scans from ELCAP and LIDC. For the training stage 61 CT images were used (36 with cancerous lung nodules and 25 without lung nodules). The system performance was tested with 45 CT scans (23 CT scans with lung nodules and 22 without nodules), different from that used for training. The results obtained show that the methodology successfully classifies cancerous nodules with a diameter from 2 mm to 30 mm. The total preciseness obtained was 82%; the sensitivity was 90.90%, whereas the specificity was 73.91%.

**Conclusions:**

The CADx system presented is competitive with other literature systems in terms of sensitivity. The system reduces the complexity of classification by not performing the typical segmentation stage of most CADx systems. Additionally, the novelty of the algorithm is the use of a wavelet feature descriptor.

## Background

Cancer refers to the abnormal growth of cells anywhere in the body; which tends to proliferate in an uncontrolled way [1]. Many cancers and the abnormal cells which compose it are further identified by the name of the tissue that the abnormal cells originated from, for example, breast cancer, lung cancer, colon cancer, prostate cancer, and so on. Lung cancer is a leading cause of death worldwide [2].

Lung cancer refers to the uncontrolled growth of abnormal cells in the lung. Typically, a computed tomography (CT) scan of the thorax is the most sensitive method for detecting lung nodules and the surrounding structures. A CT scan is a painless, noninvasive diagnostic imaging procedure which creates precise multiple images (slices) of the body structures, such as the lungs [3]. The cross-sectional images generated during a CT scan can be reformatted in multiple planes, and can generate 3D images. The national lung screening trial (NLST) has shown a relative risk reduction in lung-cancer-specific mortality of 20% and 6.7% in all-cause mortality using low dose CT screening [4].

A lung nodule is a round lesion with a diameter smaller than 3 cm. It, can be either benign (non-cancerous) or malignant (cancerous), and is found in 1 of each 100 CT scans of the chest [5]. In a CT scan, the lung cancer is observed as round white shadow nodules, therefore it is important to detect and classify those nodules for the screening and diagnosis purposes.

The likelihood that a nodule can be cancerous is about 40%, however, the risk varies considerably depending upon several factors. For example, in people with age less than 35 years, the chance that a lung nodule can be cancerous is minor than 1%, whereas the half of lung nodules in people over 50 are malignant (cancerous) [6]. When a nodule is detected on a CT scan, the radiologists must compare the current CT scan with the previous ones. If the nodule on earlier CT scans has not changed in size, shape or appearance, it is probably non-cancerous. If a lung nodule is new or has changed in size, shape or appearance, then a bronchoscopy or tissue biopsy is recommended to determine if it is cancerous.

The possibility to obtain an accurate interpretation from CT scans demands a big effort by the specialists, due to the large number of scans that are often managed and analyzed. The analysis becomes more complex when the progress of the disease is still not visually significant (early stage) [7]. For the radiologist, the process of examine a CT scan to detect lung nodules takes between 15 and 20 minutes. On the same day, the radiologist typically analyzes, at least, 45 images and this might be a fatiguing process. Therefore, different diagnosis results can be obtained by different specialists for the same scan.

There are two main computational systems developed to assist radiologists, they are: computer-aided detection (CAD) and computer-aided diagnosis (CADx) systems. CAD systems detect lesions through medical images, for example, marking conspicuous structures and sections. While CADx systems aim to measure the lesion characterization, for example, determining the malignancy and staging of the cancer [8]. CADx systems aim to improve the sensitivity, specificity, efficiency, and cost-effectiveness of lung cancer screening programs.

In this paper, we focused on the design of a CADx system that would be helpful for assisting radiologist as a second opinion to classify lung nodules and to reduce the time of the CT scan evaluation.

For the radiologist, lung nodules are usually accidentally detected in a CT scan, because they are not big enough to easily be seen. In this paper, the nodules were characterized by the computation of the texture features obtained from the gray level co-ocurrence matrix (GLCM) in the wavelet domain and were classified using a SVM with radial basis function in order to classify CT images into two categories: with cancerous lung nodules and without lung nodules.

### Related work

In the literature, several CADx approaches have been proposed for the task of classification of lung nodules using CT scans. Some of them present bibliographic reviews, for example, Li [9] and Ambrosini et al. [10], showed advances until the year of 2012.

The first reports of the use of digital computers to detect and classify lung nodules in chest radiographs occurred in 1963. Most methods consist of four steps: a) preprocessing, b) lung segmentation, c) nodule candidate detection and d) nodule classification. The classification module can differentiate malignant lesions from benign lesions using their inherent characteristics [11]. Following, a brief revision of five works about CADx systems is presented.

A methodology which uses a hybrid classification scheme was proposed by [12]. In order to determine if there are lung nodules inside the CT scans, a stage of feature extraction based on the nodule form was implemented. This causes that some blood vessels were classified as lung nodules. At the second stage, the texture features were calculated in order to discriminate the blood vessels. The approach used for classifications was a combination of SVM with a rule-based system. The CT images obtained from 3A grade hospital in Guangzhou, contains an unbalanced data set of 254 candidates regions of interest (ROI) including 50 nodules and 204 non-nodules. With the combination of these two methods a sensitivity of 84.39% was obtained.

The work in [13] describes the design and development of a two stages CADx system that can automatically detect and diagnose histological images such as CT scan of lung with a nodule into cancerous or non-nodule. In the first stage, the input image is preprocessed and the cancerous nodule region is segmented. The second stage involves in diagnosis of the nodule based on fuzzy system based on the area and the grey level of the nodule region. For the tests 40 clinical cases containing 685 slice images were used. The sensitivity obtained by the proposed method was 90%.

The diagnostic performances of artificial neural networks (ANNs) and multivariable logistic regression (LR) analysis for differentiating between malignant and benign lung nodules on CT scans is presented in [14]. The study evaluated 135 malignant nodules and 65 benign nodules. For each nodule, 4 morphological features were extracted (size, margins, contour, internal characteristics). Based on 200 bootstrap samples generated from the initial data set, 200 pairs of ANN and LR models were built and tested. The results obtained shown that ANNs had a higher discriminative performance tan LR models. The overall sensitivity for ANNs was 90% and for LR models was 86.9%.

A new approach for texture features extraction using GLCM from volumetric lung CT image is presented in [15]. The work proposed the use of 3D imaging to represent a 3D object in a more realistic way. The typical Haralicks textures features are extended in 3D and computed from volumetric data considering 26 neighbors. The optimal texture features are selected based on area under curve values of receiver operating characteristic (ROC) curve. The nodules were classified using an artificial neural network (ANN) considering the top five 3D textures and top five 2D textures features separately. For the tests 92 CT images were used. Classification using 3D texture features and 2D texture features provide 97.17% and 89.1% sensitivity respectively.

In the work proposed by [16], a system for lung nodule detection, segmentation and recognition using CT was presented. The lung area was segmented using active contours, then a masking technique was used to transfer non-isolated nodules into isolated ones. Nodules were detected using a SVM with 2D stochastic and 3D anatomical features. Four data sets were used for the tests. The first clinical data includes 13 nodules. The second group includes 6 nodules. The third group obtained from ANODE09 contains 39 nodules. Finally, the fourth group obtained from ELCAP contains 397 nodules. The overall sensitivity obtained was 89%.

In Table 1, a performance comparison of 11 recent works (including this work) related to CADx systems is shown. It should be noted that in the works presented in Table 1, different methodologies were used to create the CADx system. For example, several papers performed a segmentation task to detect nodules and the descriptors were computed in the spatial domain. Other papers perform the classification only with clinically data. However, a paper that does not require the stage of segmentation and that use the wavelet transform as a feature descriptor in a joint way was not detected in the literature. Therefore, those characteristics are the main contributions of this paper.

**Table 1:** **Performance comparison of CADx systems by sensitivity**

**Author**	**Classifier**	**Sensitivity**
Jing Z. et al. (2010) [12].	Ruled-based support vector machine	84.39%
Lee M. et al. (2010) [17].	Genetic algorithm with the random subspace method	95%
Anand S. K. V. (2010) [18].	Artificial neural network /inference and forecasting	89.6%
Kumar S A. et al. (2011) [13].	Fuzzy system	90%
Dmitriy Z. et al. (2011) [19].	Decision trees	69%
Chen H. et al. (2012) [14].	Artificial neural network and multivariable logistic regression	90%
Kumar S. A. et al. (2013) [15].	Artificial neural network	89.1%
Keshani M. et al. (2013) [16].	Support vector machine	89%
Zhang F. et al. (2014) [20].	Support vector machine and probabilistic latent semantic analysis	83%
Kuruvilla J. et al. (2014) [21].	Neural network	91.4%
Our method (2015)	Support vector machine with radial basis function	90.90%

## Methods

**Figure 1:**
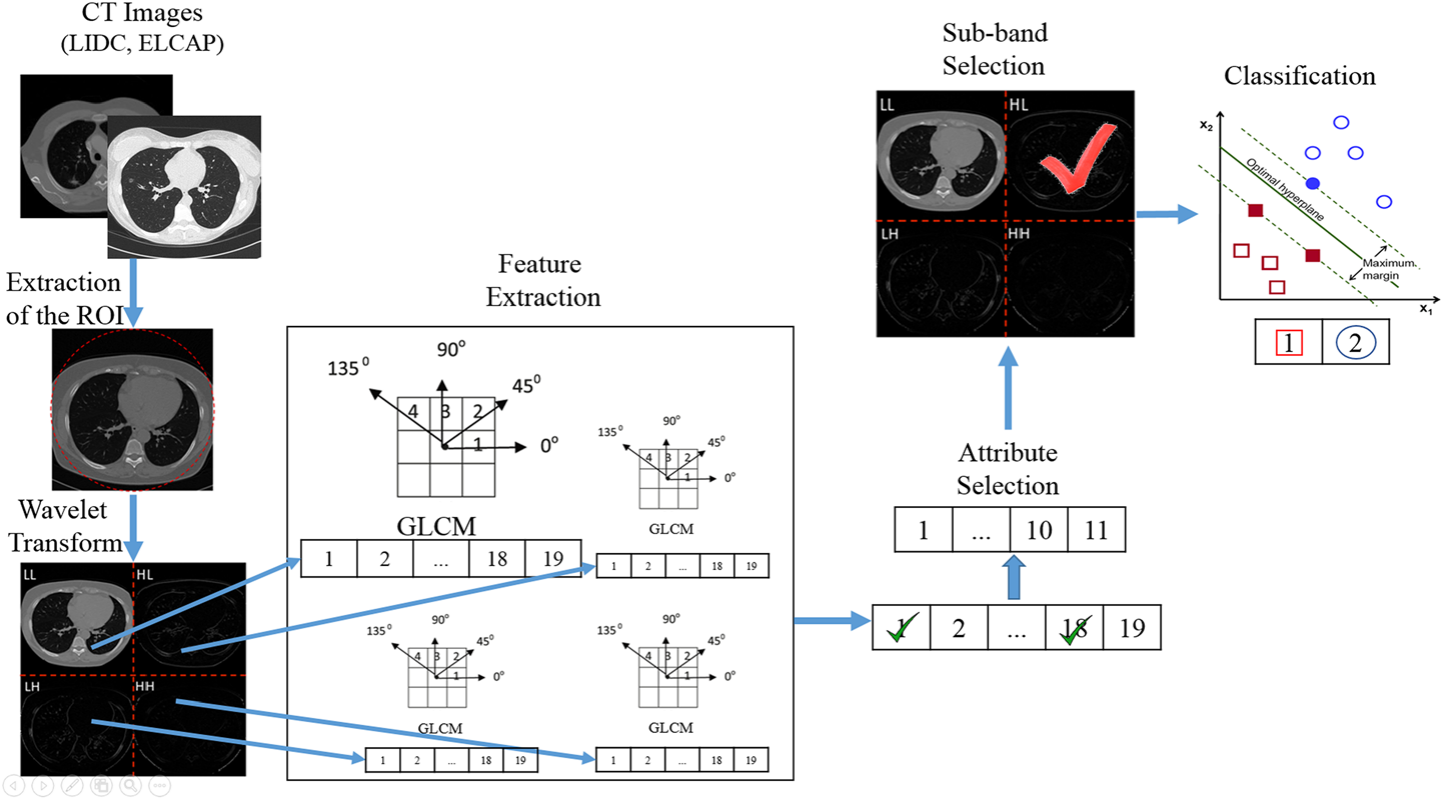
**Flow chart of the CADx system proposed.** The figure shows a detailed view of the stages followed to perform the proposed methodology. The arrows indicate the order in which each stage is performed.

The stages of the proposed methodology to design the CADx system are: 1) Extraction of the region of interest, 2) Wavelet transform, 3) Feature extraction, 4) Attribute and sub-band selection and 5) Classification. In Figure 1 the flow chart of the proposed methodology is shown.

The foremost step in medical image processing is image acquisition. For this paper, the CADx system used as an input a set of CT scans to be analyzed in order to classify lung nodules. A literature review was made in order to detect reference standard data sets that can provide the ground truth for the CADx system. One such data set was the early lung cancer action project (ELCAP) [22], the other one was the lung image database consortium (LIDC) [23].

Both databases are quite different, because the characteristics of the scanners used to obtain the CT scans are distinct. The differences of both data sets are very important in order to increment the capability of generalization of the classifier used. Evidences of works that uses both data sets with good results can be found in [24,25].

The CADx system was validated with 45 CT scans selected from the two cited public databases. The first subset contains 16 cancerous scans from the ELCAP database. The second subset contains 29 scans (7 cancerous and 22 non-cancerous) from the LIDC available in the national biomedical imaging archive (NBIA). At this stage, all the digital imaging and communication in medicine (DICOM) images were not subject to any preprocessing task.

ELCAP consists of an image set of 50 low-dose documented whole-lung CT scans for detection. The CT scans were obtained in a single breath hold with a 1.25 mm slice thickness. The database resolution is 0.5 mm × 0.5 mm and scan parameters approximately 30–40 mA. It contains a total of 397 nodules of diameter ranging from 2 mm to 5 mm [22].

In LIDC the nodules have been fully annotated by multiple radiologists. It consists of 84 CT scans, but only 58 CT scans contain nodules. The nodule diameters range from 3 mm to 30 mm. There are around 310 slices per scan, and each slice has a resolution of 512 × 512 pixels and a gray-level of 4096 HU. The pixel size ranges from 0.5 mm to 0.76 mm, and the reconstruction interval ranges from 1 mm to 30 mm. The images were acquired with several CT scanners of different manufacturers, using protocols which include low and high (40–388 mA) tube current, thin and thick (1.25-3 mm) slice thickness, 120–140 kV and various reconstruction kernels [23].

**Figure 2:**
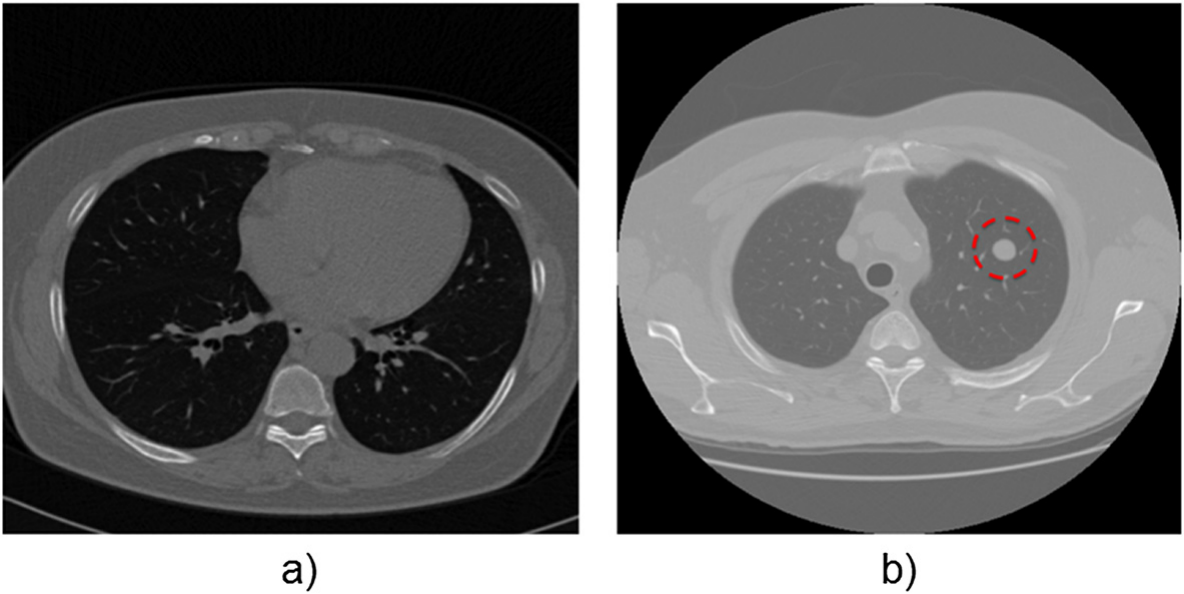
**Original CT thorax scan images: a) Without lung nodules and b) With lung nodules.** Both images were obtained from LIDC database. On the right the CT image presents a lung nodule with a diameter of 3 mm. The nodule is highlighted with a red dashed circle.

A radiologist expert validated the nodule classification. The diameters of the nodules in the CT scans selected range from 2 mm to 30 mm. An example of a CT image from LIDC with and without nodules is shown in Figure 2.

### Extraction of the region of interest

**Figure 3:**
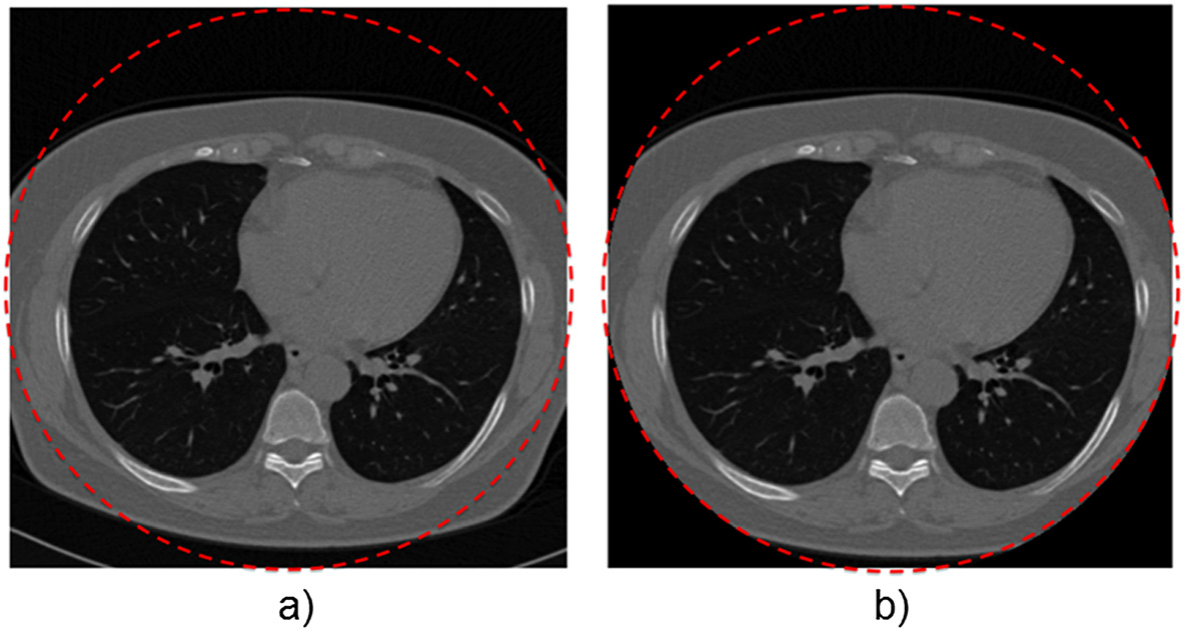
**Extraction of the ROI in a CT image: a) Original CT image and b) CT image after extraction of the ROI.** The images were obtained from two different public databases and different scanners were used for acquisition. Different forms and views can be observed. The extraction of the ROI is performed in order to eliminate the differences between images and to standardize all the CT scans. The final form is highlighted with a dashed red circle.

The CT scans obtained from LIDC and ELCAP contains several slices and differences between them. For example, some CT scans have different shapes and contain the nodule information inside a circle, this is because the CT scans were acquired from different scanners. In order to eliminate the differences between the CT images and to obtain better classification results, a ROI for each CT image was extracted. The ROI was computed using the Hough transform to approximate all the CT images to a circumference, leaving inside the circle the relevant information and making it black outside. After this stage, all the CT images preserves the important information inside the circle, as shown in Figure 3. The ROI extraction is the unique preprocessing task performed to the CT images.

### Wavelet transform

After the preprocessing stage, most systems perform the task of segmentation to separate the study region from other organs and tissues in the CT scan. For the proposed CADx system, the segmentation stage is not necessary. Instead of segmentation, the images obtained from the ROI extraction are transformed from the spatial domain to the transformed domain.

A transformation refers to the change of an image representation, for example from the spatial domain to the frequency domain. A domain transformation offers an alternative representation of an image which can reveal features difficult to detect in the original domain. The transformation is carried out in order to concentrate a great quantity of the signal energy in a few number of coefficients and to obtain, as a result, the decorrelated coefficients.

A weakness shared by several CADx systems in the feature extraction stage, is that the image is analysed at one single scale, then multilevel structures in CT images representations. Studies in the human visual system support this approach since researchers have found that the visual cortex can be modelled as a set of independent channels, each with a particular orientation and spatial frequency tuning [26]. By that, in this stage a CT image is transformed using a multiscale tool called Discrete Wavelet Transform (DWT).

The DWT is a tool which can be applied on the discrete data to obtain a multi- scale representation of the original one. From the digital point of view, the original information must be represented and delivered in an efficient way. The representation deals with the ability to capture significant information of an object of interest in a small description. The DWT allows a hierarchical decomposition of an input signal into referential signal series of low resolution and its associated detail signals [27]. The DWT offers a good representation of the high frequency components (edges) and allows representation of the image in a more compact way, since a great part of the image energy is concentrated in a small set of coefficients.

There exists a large number of wavelet families in which to search for a wavelet which will efficiently represent a signal of interest in a large variety of applications. The choice of the wavelet function depends on the application. Typically, researchers are free to select a wavelet without a reasoned justification or explanation. As a general rule, most wavelets perform well if visual verification is satisfactory for the research purposes at hand [28].

The Daubechies wavelets, are a family of orthogonal wavelets characterized by its maximal number of vanishing moments for some given support. The Daubechies wavelets can have much influence into the success of texture classification because the filter affects positively the quality of the descriptors [29]. By the above, in this paper, the well-known Daubechies db1, db2 and db4 wavelet transforms were selected. However, other orthogonal wavelet families can be used.

The transformation is obtained by convolving the columns and the rows of a CT scan with a low-pass filter (scaling function *Φ* wavelet father) and with a high-pass filter (wavelet function *Ψ* wavelet mother).

**Figure 4:**
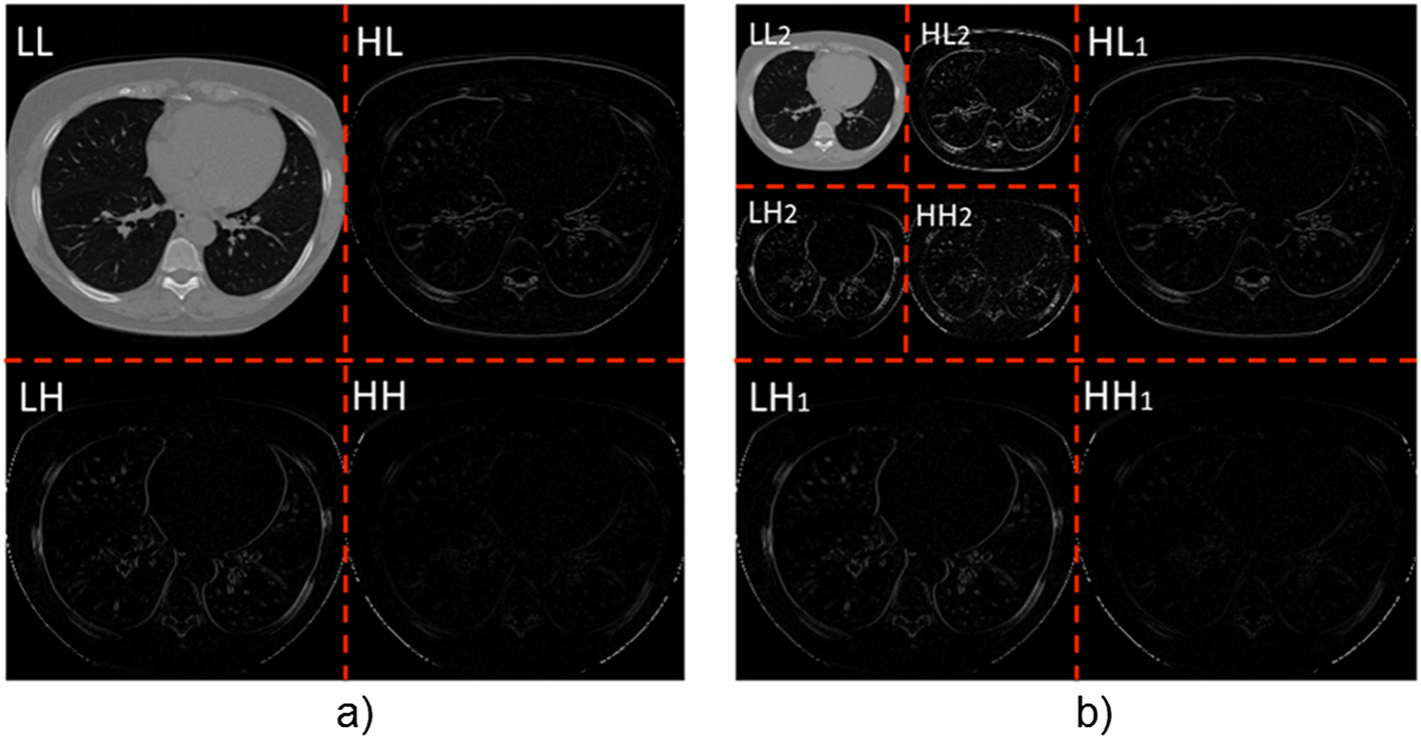
**CT image transformed from the original to the wavelet domain with the Daubechies db4 wavelet transform: a) CT image at one decomposition level and b) CT image with two decomposition levels.** Notice that the coarse sub-bands captures the information related to the lung nodules. The data from each sub-band defines a nodule candidate, and is used in the stage of feature extraction.

Let *W* and *W −1* denote the db1, db2 or db4 orthogonal DWT matrix and its inverse respectively. Then *X* = *Wx* represents the matrix of wavelet coefficients containing four frequency sub-bands (*LL*_1_, *LH*_1_, *HL*_1_ and *HH*_1_) where *L* means low and *H* means high. *LL*_1_ contains the lowest frequency coefficients or smooth information and background intensity of the image. Moreover, *LH*_1_, *HL*_1_ and *HH*_1_ contain the vertical, horizontal and diagonal detail information respectively. The DWT can be applied recursively to the resulting *LL* sub-bands for further decomposition of up to *k* levels of frequency sub-bands. For this work the values of *k = 1* and *k = 2* were computed for each CT scan, as it is shown in Figure 4.

As it can be seen from Figure 4 the *LH*, *HL* and *HH* sub-bands contain the information about the lung nodule candidates. Additionally, with the use of the DWT transform the main difficulty to distinguish true nodules from other pulmonary parenchymatous injuries or different organs and tissues is avoided.

### Feature extraction

In medical imaging, the texture can offer great information to describe the objects contained inside a CT scan. Texture plays an important role in artificial vision implementations. For example, in surface and orientation control, scene classification and object shape determination. Texture is characterized by the spatial distribution of gray levels in a neighborhood. Therefore, the texture cannot be defined by a point. The resolution in which an image is observed determines the scale in which the texture is perceived.

Texture in CT images can offer an important source of information on the state of the health of an examined organ. Diseased tissue usually has more rough or chaotic structure than the healthy counterparts, which can be characterized quantitatively for an automated diagnostic support system [30]. The quality of the extracted texture measures is of significant importance for a correct classification, especially when the difference between two different tissues becomes minor. From the medical point of view, it was observed that the texture at the edge of the lung nodules is critical in distinguishing malignant from benign nodules [31].

The gray level co-ocurrence matrix (GLCM) has been used in several works [15,32,33] extract the texture information of the lung nodules. The GLCM is the most widely used texture analysis method in biological imaging, due to its ability to capture the spatial dependence of gray level values within an image. Additionally, the characteristics typically considered by the radiologist, when classifying a nodule, are quite similar to the Haralick texture features [17], obtained from the GLCM and shown in equations 1, 2, 3, 4, 5, 6, 7, 8, 9, 10, 11.

The multiresolution analysis allows to obtain information about the candidate nodule in different scales, and then the nodule can be characterized completely from the statistical texture properties of the multiscale representation. In this stage, second order statistical texture features were extracted from the GLCM of each wavelet sub-band in order to characterize the nodules. The GLCM is a useful method to enhance the details and frequency used as an aid to define an image, is a tabulation of the frequency of different combinations of brightness values of pixels (gray tone) which occur inside an image [33]. The GLCM indicates the frequency of a group of pixels located at the same distance and direction of the displacement vector.

For each sub-band obtained after the computation of the DWT (4 for *k = 1* and 7 for *k = 2*) a set of 19 texture features defined in [13], were extracted at four different angles 0°, 45°, 90° and 135° of the GLCM. For each GLCM a quantization of 8 gray values was used.

The computed features were: autocorrelation (*Autc*, Eq. 1), entropy (*Ent*, Eq. 2), sum average (*Sav* ), sum variance (*Svar*, Eq. 3), sum entropy (*Sent*, Eq. 4), difference variance (*Diffv*, Eq. 5), difference entropy (*Diffe* ), information measure of correlation 2 (*Imc2*, Eq. 6), contrast (*Cont*, Eq. 7), dissimilarity (*Diss*, equation 8), energy (*Ener*, Eq. 9), cluster prominence (*Clpr*, Eq. 10), cluster shade (*Clsh*, Eq. 11), variance (*Var* ), inverse difference moment (*Idm* ), information measure of correlation 1 (*Imc1* ), correlation (*Corr* ), homogeneity (*Homo*) and maximum probability (*Mp*). It should be noted that only the equations of features selected with the method explained in the subsection of sub-band and attribute selection are depicted following.

1 $$ Autc=\frac{{\displaystyle {\sum}_i}{\displaystyle {\sum}_j}\left(i,j\right)p\left(i,j\right)-{\mu}_x{\mu}_y}{\sigma_x{\sigma}_y} $$

2 $$ Ent=-{\displaystyle {\sum}_i{\displaystyle {\sum}_jp\left(i,j\right) \log \left(p\left(i,j\right)\right)}} $$

3 $$ Svar={\displaystyle {\sum}_{i=2}^{2{N}_g}{\left(i- Saver\right)}^2{p}_{x+y}(i)} $$

4 $$ Sent={\displaystyle {\sum}_{i=2}^{2{N}_g}{p}_{x+y}(i) \log \left({p}_{x+y}(i)\right)} $$

5 $$ Diffv=-{\displaystyle {\sum}_{i=0}^{N_g-1}{\left(i-{\mu}_{x-y}\right)}^2{p}_{x-y}(i)} $$

6 $$ Imc2={\left(1- exp\left[-2.0(HXY2)-Ent\right]\right)}^{\frac{1}{2}} $$

7 $$ Cont={\displaystyle {\sum}_{i,j=0}^{N-1}{p}_{i,j}{\left(i-j\right)}^2} $$

8 $$ Diss={\displaystyle {\sum}_{i,j=0}^{N-1}{p}_{i,j}\left|i-j\right|} $$

9 $$ Ener=\sqrt{{\displaystyle {\sum}_{i,j=0}^{N-1}p{\left(i,j\right)}^2}} $$

10 $$ Clpr={\displaystyle {\sum}_{i.j}{\left(i+j-{\mu}_x-{\mu}_y\right)}^4p\left[i,j\right]} $$

11 $$ Clsh={\displaystyle {\sum}_{i,j}{\left(i+j-{\mu}_x-{\mu}_y\right)}^3p\left[i,j\right]} $$

where *P*_*i,j*_ is the normalized GLCM, *N*_*g*_ is the image number of rows or columns, *σ*_*x*_ and *σ*_*y*_ are the standard deviation of row *x* and column *y*, *μ*_*x*_ and *μ*_*y*_ are the mean of row *x* and column *y* respectively.

For each sub-band at one decomposition level, a set of 19 features were computed, obtaining a total of 76 features (19 for each subband *LL*, *HL*, *LH* and *HH*). The process is repeated for each angle of the GLCM, obtaining a total of 304 features (76 for each angle 0°, 45°, 90° and 135°). Finally, the process is repeated for each Daubechies filter, obtaining a total of 912 features (304 for each Daubechies filter db1, db2 and db4).

For the case of each subband of the wavelet with two decomposition levels, a set of 19 features were computed, obtaining a total of 133 features (19 for each subband *LL*_2_, *HL*_2_, *LH*_2_, *HH*_2_, *HL*_1_, *LH*_1_ and *HH*_1_). The process is repeated for each angle of the GLCM, obtaining a total of 532 features (133 for each of the four angles). Finally, the process is computed for each Daubechies filter, obtaining 1596 features (532 for each Daubechies filter).

In Table 2, the individual values of the texture features for a CT image with and without lung nodules is presented. Additionally, the values obtained when the image is rotated in an angle of 90° are presented. Observe that the values do not change with the rotation process because of the features extracted are rotation invariant. The unique difference, associated to a rotation process, corresponds to the angle in which the GLCM was calculated.

**Table 2:** **Numerical values obtained for each feature extracted of a CT image**

**Feature**	**CT with nodules**	**CT without nodules**	**CT with nodules rotated 90°**	**CT without nodules rotated 90°**
Autc	16.68	7.02	16.69	7.03
Cont	0.12	0.08	0.10	0.05
Corr	0.97	0.97	0.97	0.98
Clpr	230.76	116.87	231.67	117.21
Clsh	−28.14	12.74	−27.97	12.85
Diss	0.08	0.07	0.07	0.05
Ener	0.29	0.40	0.30	0.41
Ent	1.44	1.29	1.42	1.23
Homo	0.96	0.96	0.96	0.97
Mp	0.39	0.56	0.39	0.56
Svar	16.62	6.99	16.62	6.98
Sav	7.57	4.43	7.58	4.43
Var	47.94	18.79	47.91	19.03
Sent	1.37	1.22	1.37	1.19
Diffv	0.12	0.08	0.10	0.05
Diffe	0.28	0.27	0.25	0.20
Imc1	−0.80	−0.76	−0.81	−0.83
Imc2	0.92	0.89	0.92	0.90
Idm	0.99	0.99	0.99	0.99

### Attribute and sub-band selection

Feature or attribute selection is arguably one of the most crucial steps in the pattern recognition system design cycle, because it allows to automatically search for the best subset of attributes in the feature vector. In order to design an efficient classification system, it is important to select features that are the most effective in capturing the salient differences between the two classes described (with cancerous nodules and without nodules). In order to reduce the possibility of overfitting during the classification step, it was necessary to reduce the dimensionality of the feature vector [34]. Additionally, this stage allows to reduce the training time because less data means that algorithms train faster.

After the computation of all the 19 statistical texture features, an analysis to measure the relevance of each feature in each wavelet sub-band was carried out. The goal was to reduce the feature set and this task was made using the Waikato Environment for Knowledge Analysis (WEKA) software. Weka contains a collection of visualization tools and algorithms for data analysis and predictive modelling. Particularly, for this stage, the select attributes panel of WEKA was used.

In WEKA the method by which a subset of attributes are assessed is called the attribute evaluator. For this paper, the method selected was *CfsSubsetEval* which values subsets that correlate highly with the class value and low correlation with each other. The best first, genetic and greedy stepwise search methods were used to define the structured way in which the search space of possible attribute subsets is navigated based on the subset evaluation.

The analysis performed with the three algorithms resulted in a ranking list about the importance of each feature. After that, an analysis was performed in order to detect the most repeated important features of each algorithm. The analysis results in a recommendation to reduce the feature vector from 19 to 11 attributes which are shown in equations 1, 2, 3, 4, 5, 6, 7, 8, 9, 10, 11.

After the stage of attribute selection, a sub-band selection is performed for each of the two wavelet decomposition levels in order to reduce more the computation time. As was stated in the feature extraction section, the feature vector is computed for each sub-band with four different angles and wavelet filters. However, even when the feature vector was reduced from 19 to 11 features, a lot of features most be computed for each wavelet sub-band.

The goal of sub-band selection is to detect the sub-band that better compacts and represents the information contained in the CT. For each wavelet sub-band the attribute selection stage allows detecting the most important features globally, with the sub-band selection the analysis is made locally.

The WEKA software with the same parameters used at the attribute selection stage was used to perform the sub-band selection. The results obtained for the wavelet first and second level of decomposition are shown together with the results obtained with the classifier in Tables 3 and 4 respectively.

**Table 3:** **Results of nodule classification at wavelet first decomposition level**

**Base**	**Angle**	**Sub-band**	**Features**	**Specificity**	**Sensitivity**	**Preciseness**
Db1	0°	LH	Autc-Clsh	82.60%	63.63%	73.33%
Db2	0°	LH	Clsh-IMC2	86.95%	45.45%	66.66%
Db4	0°	HH	Autc-Diss	60.86%	95.45%	77.77%
Db1	45°	LH	Autc-Clsh	86.95%	59.09%	73.33%
Db2	45°	LH	Autc-IMC2	69.56%	68.18%	68.88%
Db4	45°	LH	Autc-IMC2	69.56%	90.90%	80%
Db1	90°	LH	Clpr-Clsh	**73.91%**	**90.90%**	**82.22%**
Db2	90°	LH	Clpr-Svar	82.60%	59.09%	71.11%
Db4	90°	HH	Autc-Diffv	52.17%	**100%**	75.55%
Db1	135°	LH	Clpr-Sent	**86.95%**	72.72%	80%
Db2	135°	LH	Clsh-Diffe	**86.95%**	72.72%	80%
Db4	135°	HH	Autc-Cont	65.21%	90.90%	77.77%

The bold data represent the best value obtained.

**Table 4:** **Results of nodule classification at wavelet second decomposition level**

**Base**	**Angle**	**Sub-band**	**Features**	**Specificity**	**Sensitivity**	**Preciseness**
Db1	0°	HH2	Clpr-Ener	68.18%	68.18%	71.11%
Db2	0°	LL	Autc-Sent	36.36%	91.30%	64.44%
Db4	0°	LL	Autc-Ent	36.36%	**95.45%**	65.90%
Db1	45°	LL	Clsh-Ener	82.60%	68.18%	**75.55%**
Db2	45°	LL	Autc-Ener	73.91%	63.63%	68.88%
Db4	45°	LL	Autc-Sent	82.6%	59.09%	71.11%
Db1	90°	LL	Autc-Ener	86.95%	50%	68.88%
Db2	90°	LL	Autc-Ent	47.82%	95.45%	71.11%
Db4	90°	LL	Autc-Ent	54.16%	71.42%	62.22%
Db1	135°	LL	Autc-Ener	65.21%	63.63%	64.44%
Db2	135°	LL	Autc-Ent	60.86%	77.27%	68.88%
Db4	135°	HH	Autc-Sent	**90.90%**	56.21%	73.33%

The bold data represent the best value obtained.

### Classification

The pattern classification is defined as the task to categorize any object within a given category called class. For this paper, the classification stage was made using a support vector machine (SVM). The SVM was developed by Vapnik to solve classification problems. The current version of SVM for regression was developed in the AT&T laboratories by Cortes and Vapnik in 1995 [35]. The theoretical characteristics of SVM are typically defined for classification problems with two different classes.

By the above, in order to train the SVM, a combination of two different features from the 11 feature vector obtained were tested. In order to obtain the best two significant features exhaustive combinations of the 11 features of each scale and filter was tested. The selection was made by a ranking of the features using an independent evaluation criterion of the absolute value of two-sample t-test with pooled variance.

The set of features was used in combinations of two features to enhance the relation size/dimensionality, to reduce the measurement, storage and computation costs and to avoid the curse of dimensionality. The curse of dimensionality explains that increasing the dimensionality of the problem by adding new features would actually degrade the performance of the classifier [36]. The work of [37] suggest that it is much harder to find patterns from many weak than from few strong informative features. By the other hand several authors suggest how to obtain the ideal size of the feature set to obtain the optimal performance of a classifier. For example, for a feature set with a dimensionality of 10, then 842,000 samples are required, for classification purposes. Many realistic study designs will typically estimate substantially suboptimal patterns and may have low probability of statistically significant validation results. Additionally, several works can be found in which a good classification rates were obtained using only two features [36,38].

Additionally, in this stage, the pairs of texture features were plotted to test if the lung nodule classification can be performed with a linear SVM. Since typically, the features of interest cannot be linearly separable, instead of fitting a non-linear function, we decide to use a kernel. The kernel used was a radial basis function (RBF) defined in Eq. 12.

12 $$ K\left({x}^t,x\right)=C\cdot exp\left[\frac{-{\left\Vert {x}^t-x\right\Vert}^2}{\sigma^2}\right] $$

where *xt* is the center, *x* is the input feature, *σ* smoothed the Gaussian thus reduces the variance (a parameter typically given by the user) and *C* is defined as a penalty factor which allows controlling the system overlearning. In this stage, it is critical to select a proper penalty factor value. If the factor is too large, then a high penalty of non-separable points is obtained, then, many support vectors need to be stored and the algorithm overfits. By the other hand, if the value is too small, then, an underfitting is obtained [35].

The SPIDER toolbox of Matlab was used to accurate compute the value of *σ*. The toolbox is an object-oriented environment for machine learning in Matlab. The centroids were calculated using WEKA software with the k-means clustering algorithm which groups data according to the average of each feature.

**Figure 5:**
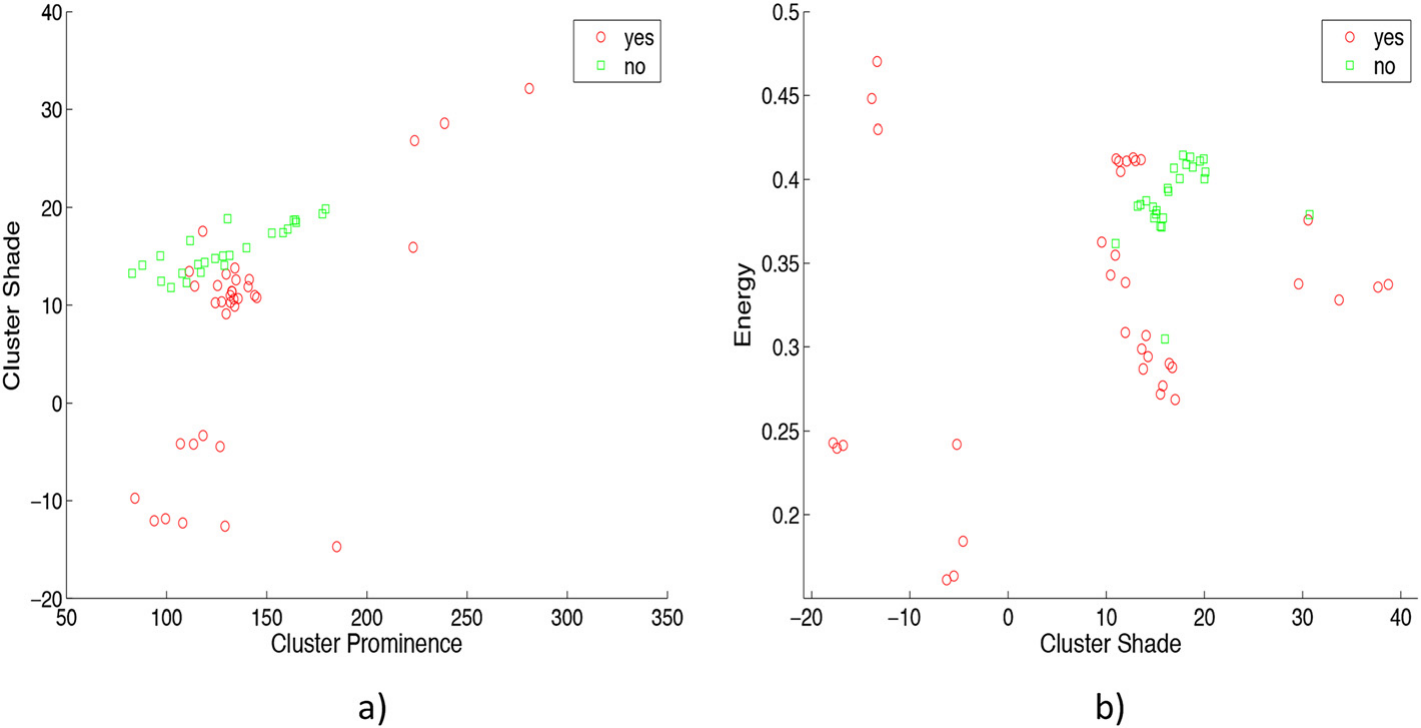
**Vectors of the SVM: a) Cluster prominence and cluster shade features and b) Cluster shade and energy features.** It should be noted that features in the set are disjoints. Then, the RBF is a suitable kernel to create the hyperplane of separation for the correspondent SVM.

The balanced data set of 45 CT scans was randomly split into training and testing sets to validate the classifier. In order to train the SVM-RBF a set of 61 CT images were used, 36 CT images with cancerous lung nodules (yes) and 25 CT images without lung nodules (no). In Figure 5 a plot of the vectors for each wavelet decomposition level is shown. The figure shows two different combinations of features. Figure 5a corresponds to the first decomposition level and Figure 5b corresponds to the second decomposition level.

**Figure 6:**
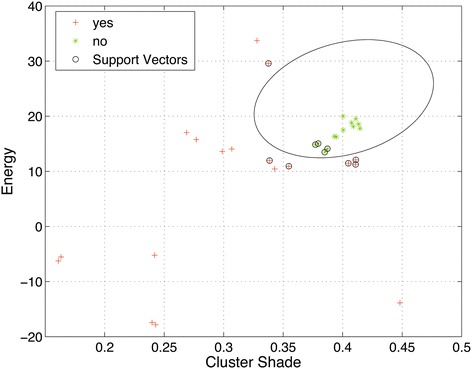
**Plot of trained vectors: Cluster prominence and cluster shade features.** The plot shows the class separation, the hyperplane and its correspondent support vectors. The green dots are the data without lung nodules presented in the image and red dots corresponds to the data with lung nodules.

For all the CT scans two different statistical texture features were computed to obtain the support vectors, the optimal hyperplane, the penalty factor and the correspondent centroids. The SVM-RBF training step was performed using those data. In Figure 6 the plot with trained support vectors for cluster shade and energy features is shown. A CT scan is classified into one of two groups: 1) With lung nodules (cross symbols) and 2) Without lung nodules (circle symbols), with this output the classification stage is finalized.

## Results and discussion

The ability of the system to classify real cancerous lung nodules inside a CT scan was evaluated and contrasted by the Professional Technical in Radiology Antonio Estrada Barrientos who has several years of experience working for the “Center of Advanced Imaging SC” in Ciudad Juarez, Mexico.

The CADx system was trained with a SVM-RBF using a total of 61 images, 36 with cancerous lung nodules and 25 without lung nodules. Furthermore, the system was tested and validated on a clinical data set, different from that used in the training stage, of 45 thoracic CT scans (each scan contains at least 200 images, involving more than 9,000 CT slices) which contains 23 CT scans with lung nodules and 22 CT scans without nodules.

Four possibilities were used to compare the results obtained: False Positive (FP) which indicates a disease when in fact does not exists, False Negative (FN) which indicates no disease when in fact does exists, True Positive (TP) which indicates a real disease and True Negative (TN) which indicates no disease. These four possibilities can be summarized in a 2 × 2 contingency table in order to compare and analyze the results obtained with the proposed algorithm and those results obtained by the radiologist. In Table 5 the contingency matrix is shown. The Table 5 corresponds to the computation of the contingency table for the Daubechies db1 wavelet transform with one decomposition level and 90° of the GLCM. For the contingency matrix, two false negatives (FN) and six false positives (FP) were obtained.

**Table 5:** **Contingency table obtained from the CADx system for the db1 LH sub-band and GLCM at 90°**

**With nodules**		**Without nodules**		**Total**
True positive	20	False positive	6	26
True negative	17	False negative	2	19
Total	37		8	45

**Figure 7:**
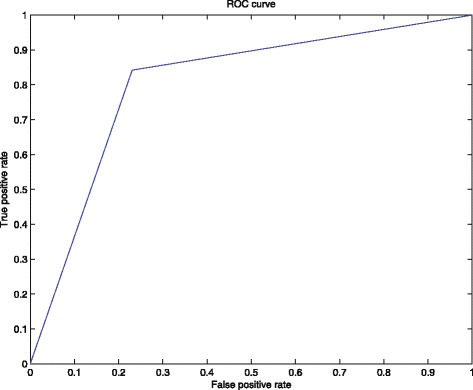
**The ROC curve obtained for the SVM model.** The plot was built with perfcurve matlab function using the information shown in Table 5. The value obtained for the AUC was 0.805.

In order to obtain information on how accurately the SVM distinguishes subjects with different outcomes (i.e., a CT image with a cancerous nodule or without nodules), the receiver operating characteristic (ROC) curve was computed. The ROC curve is a popular and powerful tool to assess discrimination for binary outcomes. The curve is created by plotting the true positive rate against the false positive rate at various threshold settings [39]. The ROC curve obtained for the SVM using the information in Table 5 is shown in Figure 7.

After the computation of the ROC curve, the area under curve (AUC) was obtained. The AUC measures the discriminatory ability of the SVM, where a value of 1.0 perfect discriminatory power and a value of 0.5 indicates no discriminatory ability [40]. The AUC value obtained was 0.805.

**Figure 8:**
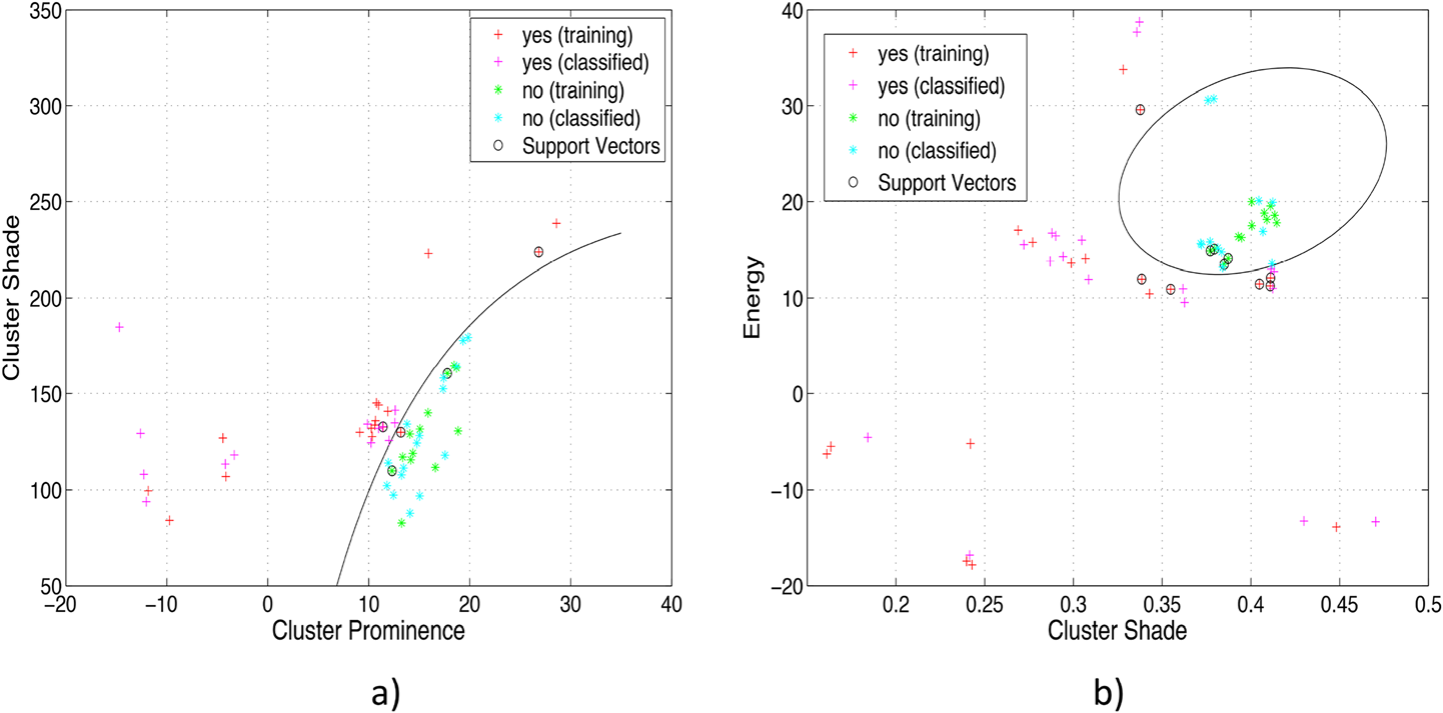
**Plot of classification results with the SVM: a) Cluster prominence and cluster shade features and b) Cluster shade and energy features.** The plot shows the results obtained with a pair of features for the classification stage. 45 CT images were used to test the SVM created, the correct and incorrect data classification can be observed in a visually way.

In Figure 8 the results of two different tests are shown, where 8a corresponds to the first decomposition level of the wavelet and 8b corresponds to the second decomposition level. The parameters used were *C = {1, 1e*^*1*^*, 1e*^*2*^*, 1e*^*3*^*, 1e*^*4*^*}* and *σ* = *{*4*,* 32*}*.

Diagnosis tests should ideally have a sensitivity and specificity as close as possible to 100%. The specificity of a test, defined by Eq. 13, indicates the probability of obtaining a negative result when the individual does not have the disease. The sensitivity, defined by Eq. 14, of a diagnostic test is the probability of obtaining a positive result when the individual has the disease. The preciseness, computed with Eq. 15, is the proportion of valid results obtained from all the tests performed.

13 $$ sensitivity=\frac{TP}{TP+FN} $$

14 $$ specificity=\frac{TN}{TN+FP} $$

15 $$ preciseness=\frac{TP+TN}{TP+FP+TN+FN} $$

In Tables 3 and 4 the results obtained for all the tests made for the first and second wavelet decomposition levels with four different angles of the GLCM are shown. The first column shows the wavelet used, the second column shows the angle of the GLCM, the third column represents the sub-band selected; the fourth column presents the couples of features chosen after attribute selection stage. Finally, the columns fifth, sixth and seventh shows specificity, sensitivity and preciseness respectively. The best value obtained is highlighted in bold.

For the case of the contingency matrix shown in Table 5 the best sub-band was *LH* and better texture feature combinations was *Clpr* (Eq. 10) and *Clsh* (Eq. 11). Also, the better specificity, sensitivity and preciseness obtained was 73.91%, 90.90% and 82.22%, respectively.

All the images (more than 9000) inside the 45 CT scans were analyzed for the stage of testing the classifier. The classification was made after all the images of each scan were tested. For example, an array of size equals to the total number of images in the CT scan was created. Even when one or more nodules appears inside the image, the correspondent position of the array was marked as cancerous. By the above, only one nodule was counted obtaining a total of 16 for ELCAP and 7 for LIDC. The same occurs for the 22 non-cancerous images, only one count was made after testing all the images.

The nodule diameters range from 2 mm to 30 mm. The major percentage of errors were obtained for smaller nodules. For the case of ELCAP always the 16 nodules were detected in an adequate way. With the LIDC data set 5 or 6 nodules were detected correctly. The erroneous data was obtained always with LIDC data set, and the correspondent values can be verified in Table 5.

The results obtained at the experimentation stage demonstrated the ability of the system to classify lung nodules. As shown in Table 1, making a comparison against the other methods in the literature, the method presented in this paper is competitive, taking into account the information shared about sensitivity, even when the information about TP, TN, FP and FN is not always presented in the other works, by that it is difficult to offer a complete real comparison against all the works.

## Conclusions

In this paper, a CADx system to classify lung nodules using features computed from the GLCM of a Daubechies db1, db2 and db4 wavelet transform and support vector machines with radial basis as classifier was proposed. The novelty of the paper is the elimination of the typical structure segmentation stage, this is because the detection of candidate lung nodules is carried out by means of a wavelet transform. Another novelties of the system are the use of wavelet features to describe the lung nodules and that the only preprocessing stage performed is the extraction of a ROI.

The results obtained were favorable, texture feature extraction and SVM-RBF as a classifier indicate whether the CT scan has lung nodules or note. The better results were obtained with the angles of 90° or 135° of the GLCM with one and two decomposition levels.

The ability and the certainty of the system to classify lung nodules inside a CT scan was validated by a professional technical in Radiology Antonio Estrada Barrientos. The methodology was trained with 61 CT images (36 CT images with lung nodules and 25 CT images without lung nodules) and validated on a clinical data set, different from that used in the training stage, of 45 thoracic CT examination files (involving about 9,000 CT slices) which contains 23 CT examination files with lung nodules and 22 CT scans without nodules.

The results show that the methodology can successfully classify nodules from 2 mm to 30 mm in diameter. At the test stage sometimes the algorithm marked a nodule in some files that the radiologist did not see anything, so it is possible that there were really pulmonary nodules. In the files that were obtained from the ELCAP database there was a classification of 100% of nodules, detecting pulmonary nodules with a diameter smaller than 4 mm, while for the images obtained from LIDC database the rate was about 81%. As it was stated in the materials and methods section, all the CT scans from ELCAP contain lung nodules while the scans from LIDC contain diseases but not in all the nodules are presented. The methodology is competitive compared with other works presented in the literature.

In the future the methodology proposed will be tested using different classifiers such as neural networks, random forest or decision trees and with other transforms such as contourlets, edgelets and bandelets. Additionally, it will be important to train and test the system using CT scans with benign nodules.

## Abbreviations

AUC: Area under curve
ANN: Artificial neural network
CAD: Computer-aided detection
CADx: Computer-aided diagnosis
CT: Computed tomography
DICOM: Digital imaging and communication in medicine
DWT: Discrete wavelet transform
FP: False positive
FN: False negative
GLCM: Gray level co-ocurrence matrix
LR: Logistic regression
LIDC: Lung image database consortium
NBIA: National biomedical imaging archive
NLST: National lung screening trial
RBF: Radial basis function
ROC: Receiver operating characteristic
ROI: Region of interest
SVM: Support vector machine
TN: True negative
TP: True positive
WEKA: Waikato environment for knowledge analysis
